# Beavers indicate metal pollution away from industrial centers in northeastern Poland

**DOI:** 10.1007/s11356-014-3769-8

**Published:** 2014-11-05

**Authors:** Aleksandra Giżejewska, Anna Spodniewska, Dariusz Barski, Julien Fattebert

**Affiliations:** 1 Department of Pharmacology and Toxicology, Faculty of Veterinary Medicine, University of Warmia and Mazury in Olsztyn, 10-957 Olsztyn, Poland; 2 School of Life Sciences, University of KwaZulu-Natal, Westville Campus, Durban, 4000 South Africa

**Keywords:** *Castor fiber*, Contamination, Cadmium, Copper, Lead, Zinc, Liver, Kidney

## Abstract

Heavy metals are persistent environmental contaminants, and wild animals are increasingly exposed to the harmful effects of compounds of anthropogenic origin, even in areas distant from industrial centers. We used atomic absorption spectrometry to determine levels of cadmium (Cd), lead (Pb), copper (Cu), and zinc (Zn) in liver and kidney of wild Eurasian beavers (*Castor fiber*) in Poland. Cd concentrations in liver (0.21 ± 0.44 μg/g) and in kidney (2.81 ± 4.52 μg/g) were lower in juvenile than in adult beavers. Pb concentrations in liver (0.08 ± 0.03 μg/g) and kidney (0.08 ± 0.03 μg/g) were similar among all individuals, while both Cu and Zn levels were higher in liver (Cu 9.2 ± 4.5 μg/g; Zn 35.7 ± 3.5 μg/g) than in kidney (Cu 3.7 ± 1.1 μg/g; Zn 21.5 ± 2.7 μg/g). Cu levels also differed between juveniles and adults. We reviewed the literature reporting metal concentrations in beavers. Our results indicate metal contamination in beavers away from important industrial emission sources and suggest the natural environment should be regularly monitored to ensure their levels are below recommended, legal values.

## Introduction

Wild animals are exposed to environmental contaminants of both natural and anthropogenic origin and are suitable bioindicators of environmental pollution (O’Brien et al. 1993; Mason and Stephenson 2001; Ansara-Ross et al. 2013; Petkovšek et al. 2014). Concentrations of toxic compounds in body tissues are determined by the degree of exposure, the compound’s bioavailability and half-life, as well as the type of tissue (Nordberg et al. 2011a). Pollutants can be transported over significant distances by air or water, and they are found even in regions situated remotely from industrial centers (Ansara-Ross et al. 2013; Jerez et al. 2013). Therefore, due to the rapid development and wide diversity of human activities, animals are increasingly exposed to harmful effects of compounds of anthropogenic origin (O’Brien et al. 1993). With growing public awareness of environmental pollution, regular monitoring of pollutants in the environment is essential to ensure levels of pollutants are below harmful or recommended, legal values (Nasreddine and Parent-massin 2002; European Commission 2006). Such monitoring should be carried out not only in industrialized areas but also in natural and agricultural ecosystems away from the source of emissions (Nasreddine and Parent-massin 2002; Szkoda et al. 2012).

Metals include toxic elements such as cadmium (Cd) and lead (Pb), as well as essential elements in which toxicity is determined by the dose, as copper (Cu) and zinc (Zn). Although these four elements occur naturally in the environment, they are supplied in large quantities through domestic and industrial pollution (Bjerregaard and Andersen 2011). Heavy metals are also persistent environmental contaminants whose physiochemical properties contribute to their mobility in the natural environment, posing a significant threat for living organisms and ecosystems (Bjerregaard and Andersen 2011).

Previous studies reported essential and toxic metals in beaver (family: Castoridae) tissues obtained from industrial areas, generally regarded as polluted (Hillis and Parker 1993; Zalewski et al. 2012), agricultural regions (Fimreite et al. 2001), and natural ecosystems (Wren 1984; Zalewski et al. 2012; Giżejewska et al. 2014). Beavers are semi-aquatic, territorial herbivorous rodents feeding on the bark, shoots, and leaves of a wide variety of woody plants including willow (*Salix* sp.) and aspen (*Populus tremuloides*), as well as on non-woody terrestrial and aquatic plants (Haarberg and Rosell 2006; Krojerová-Prokešová et al. 2010). Beavers’ catholic diet and sedentary behavior make them suitable bioindicators of local environmental pollution. In Poland, the status of Eurasian beavers (*Castor fiber*) as a “partially protected” species (Polish Minister of the Environment 2011) contributed to rapid population growth. It is currently estimated that 78,000 beavers occur in the country, and beaver populations continue spreading to new areas, including near human settlements, where they are likely to get exposed to anthropogenic pollutants (Flis 2013).

In this study, our aim was to use the Eurasian beaver as a bioindicator organism (i) to assess trace element contents in a region away from major industrial centers and (ii) to evaluate the degree of contamination against legal Cd and Pb levels recommended by the European Commission in farm animal edible tissues (European Commission 2006) with regard to the lack of an official threshold for wildlife (Giżejewska et al. 2014). Specifically, we measured concentrations of Cd, Pb, Cu, and Zn in liver and kidney of wild beavers. We predicted (i) metal concentrations to differ between liver and kidney according to element-specific metabolism, (ii) no sexual difference in metal concentrations in this non-dimorphic species, and (iii) higher metal concentrations in adult than in juvenile individuals because of bioaccumulation processes. We also present a review of the literature reporting metal concentration in beavers. We compared our results with previous research, and we discuss the implications for future use of beavers as bioindicators for the monitoring of metal pollution.

## Material and methods

Eurasian beavers were captured in Wiżajny municipality (22° 52′ E; 54° 22′ N), Warmia and Mazury Region, northeastern Poland, in Summer 2012. Warmia and Mazury is a traditional agricultural region with extensive forests and numerous lakes. The region is an important recreational center for local and international tourists who seek activities in a natural environment. The sampling site was away from a major industrial complex, which could putatively be a source of pollutants in their vicinity. Animals were collected upon approval of the Regional Nature Conservation Authority (Opinion No. RDOŚ-28-OOP-6631-0007-638/09/10/pj 2010) and the Local Ethics Committee (Opinion No. 11/2010).

All beavers were collected during daytime with a net and transported to the laboratory in cages specially adapted for this purpose (Zalewski et al. 2012; Giżejewska et al. 2014). All animals were healthy and in good physical condition at the time of capture. Their age was estimated based on body weight (Rosell et al. 2010), and they were classified into two age classes: (i) juveniles, 2 months old, and (ii) adults, >24 months old (Table 1). Sex was determined based on the color of anal gland secretions (Schulte et al. 1995). Beavers were anesthetized with xylazine and ketamine in doses appropriate to body weight, and body measurements taken.

**Table 1:** Biometric data of 10 Eurasian beavers from Wiżajny municipality, northeastern Poland, 2012

Individual	Sex	Age class	Body weight (kg)	Body length (cm)
M1	♂	Juvenile	2.5	62
M2	♂	Juvenile	2.8	62
F3	♀	Juvenile	2.8	64
F4	♀	Juvenile	2.7	60
M5	♂	Adult	18.6	116
M6	♂	Adult	22.6	114
F7	♀	Adult	17.3	115
F8	♀	Adult	21.1	117
F9	♀	Adult	22.7	118
F10	♀	Adult	22.0	121

Then, beavers were sacrificed by decapitation under full anesthesia. Liver and kidney samples of c. 15 g each were collected and stored in polyethylene bags at −25 °C until analysis (Zalewski et al. 2012; Giżejewska et al. 2014). Each sample was homogenized and two subsamples were prepared from each individual organ and analyzed twice each as replicates. All tissue samples were mineralized in the muffle furnace at 450 °C for 24 h. Ashes were dissolved in 1 M HNO_3_ and deionized water was added to 25 ml (Łuczyńska et al. 2009). We performed graphite furnace atomic absorption spectroscopy to determine Cd and Pb concentrations and flame atomic absorption spectroscopy to determine Cu and Zn concentrations (Whiteside and Milner 1984) using an iCE 3000 Series spectrometer (Thermo Scientific, UK).

Analysis for each element was performed based on calibration curves plotted from working standards. For calibration, we used commercial stock solutions (1000 mg/l) of analytical grade for all four elements (J.T.Baker®). Working standards were prepared by dissolving stock solutions with 0.1 M HNO_3_ and deionized water. Calibration ranges were 0.0005–0.004 μg/g for Cd, 0.001–0.01 μg/g for Pb, 0.05–0.8 μg/g for Cu, and 0.1–0.8 μg/g for Zn, respectively. Limits of detection were 0.002 μg/g for Cd, 0.001 μg/g for Pb, and 0.05 μg/g for Cu and Zn. Reagent blanks and control samples containing all reagents were prepared and run in duplicate. Quality control of analytical measurements was performed using certified reference material (BCR-185R bovine liver, IRRM, Belgium). Each working solution was analyzed in duplicate and calibration curves were periodically verified. Recoveries of all four elements were within the range 86–109 % and relative standard deviations were below 10 %.

Metal concentrations were expressed in micrograms per gram of wet weight, with precision consistent with limits of quantitation—0.001 μg/g for Cd and Pb and 0.1 μg/g for Cu and Zn. Metal concentrations were contrasted between organs among all beavers using a paired *t* test. Concentrations in beaver livers and kidneys were compared between age or sex classes using a non-parametric Mann-Whitney *U* test. Pair-wise comparisons between element concentrations in each organ were performed using a Spearman rank correlation analysis. We report arithmetic means, standard deviations (SD), median, and range. Where necessary, data were transformed to meet statistical assumptions. Significance level was set at *p* < 0.05, and all statistical analyses were run using R version 3.0.0 (R Core Team 2013).

## Results

We captured six female (four adults, two juveniles) and four male (two adults, two juveniles) beavers (Table 1). Mean concentrations of Cd, Pb, Cu, and Zn determined in liver and kidney samples are presented in Table 2 and compared with the literature (Table 3). Higher concentrations in liver than in kidney were found for Cu (*t* = −4.534; *df* = 9; *p* = 0.001) and Zn (*t* = −19.826; *df* = 9; *p* < 0.001). Additionally, Zn levels were significantly correlated to Cd levels in kidney (*n* = 10, *r*_*s*_ = 0.939; *p* < 0.001).

**Table 2:** Summary statistics of the concentration (μg/g wet weight) of four metals (Cd, Pb, Cu, and Zn) in liver and kidney of 10 Eurasian beavers, NE Poland, 2012

Metal	Mean ± SD	Median	Range	Maximum legal level^a^
Liver				
Cd	0.21 ± 0.44	0.06	0.03–1.44	0.5
Pb	0.08 ± 0.03	0.06	0.05–0.12	0.5
Cu	9.2 ± 4.5	6.9	5.2–16.4	–^b^
Zn	35.7 ± 3.5	36.2	31.4–40.6	–^b^
Kidney				
Cd	2.81 ± 4.52	1.37	0.02–14.88	1.0
Pb	0.08 ± 0.03	0.07	0.05–0.14	0.5
Cu	3.7 ± 1.1	3.5	3.0–3.8	–^b^
Zn	21.5 ± 2.7	20.2	19.0–27.9	–^b^

^a^Maximum levels set by the European Commission (2006) for contaminants of toxic elements in food of animal origin

^b^No regulation

**Table 3:** Average concentrations (μg/g wet weight) of Cd, Pb, Zn, and Cu in liver and kidney of beavers (family: Castoridae) given in the literature

Species	Liver				Kidney				Sites	Reference
	Cd	Pb	Cu	Zn	Cd	Pb	Cu	Zn		
*C. fiber*	0.21	0.08	9.2	35.7	2.81	0.08	3.7	21.5	Unpolluted	This study
*C. fiber*	0.88	0.11	–	–	7.93	0.06	–	–	Unpolluted	Giżejewska et al. (2014)
*C. fiber*	0.58	0.19	4.04	23.73	7.10	0.13	2.73	25.23	Former military airport	Zalewski et al. (2012)
*C. fiber*	0.22	0.14	4.40	27.52	2.44	0.09	3.15	27.05	Unpolluted	Zalewski et al. (2012)
*C. fiber*	1.03	–	2.8	27.66	10.25	–	2.17	23.11	Unpolluted	Fimreite et al. (2001)
*C. canadensis*	2.3	2.7	8	80.8	18.8	3.4	9.3	99.3	15 km from ore smelter	Hillis and Parker (1993)
*C. canadensis*	–	1.4	–	–	–	2.1	–	–	90 km from ore smelter	Hillis and Parker (1993)
*C. canadensis*	0.19	–	2.9	29.6	1.44	–	3	25.4	175 km from ore smelter	Wren (1984)

**Fig. 1:**
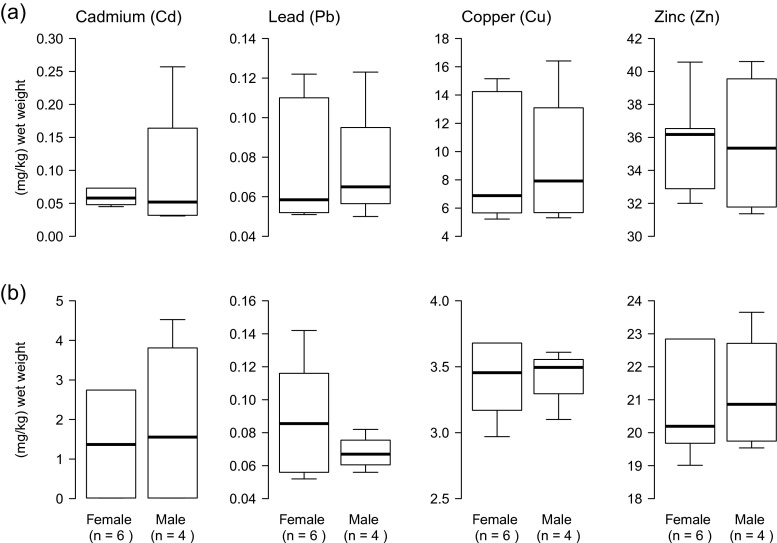
Concentrations (μg/g wet weight) of Cd, Pb, Cu, and Zn in **a** liver and **b** kidney of six female and four male Eurasian beavers, NE Poland, 2012. No statistical significant difference in metal concentrations between sex classes was found (Mann-Whitney *U* test, all *p* > 0.5). *Box* and *whisker plots* show median (*horizontal line* within *box*), 25 and 75 % percentiles (*box*), 1.5 interquartile range (*whiskers*). For clarity, extreme outliers are not shown

**Fig. 2:**
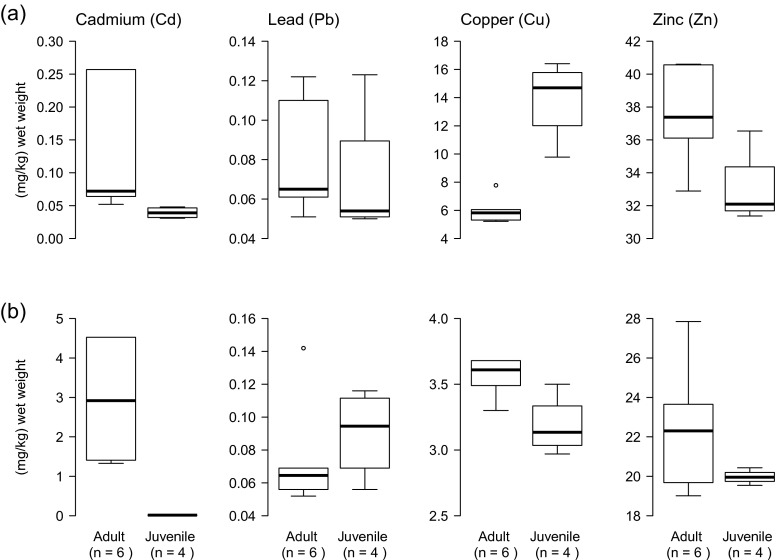
Concentrations (μg/g wet weight) of Cd, Pb, Cu, and Zn in **a** liver and **b** kidney of six adult and four juvenile Eurasian beavers, NE Poland, 2012. Significant statistical differences in metal concentrations between age classes were found in liver Cd (*U* = 0.0; *Z* = −2.452; *p* = 0.014) and Cu (*U* = 0.0; *Z* = 2.452; *p* = 0.014) and in kidney Cd (*U* = 0.0; *Z* = −2.452; *p* = 0.014) and Cu (*U* = 2.0; *Z* = −2.025; *p* = 0.043). *Box* and *whisker plots* show median (*horizontal line* within *box*), 25 and 75 % percentiles (*box*), 1.5 interquartile range (*whiskers*), and statistical outliers (*open circles*). For clarity, extreme outliers are not shown

We found no differences between female (*n* = 6) and male (*n* = 4) beavers for any of the four metal concentrations in liver or kidney (Fig. 1). Contrastingly, we found significant differences in Cd and Cu concentrations between adult and juvenile beavers (Fig. 2). Cadmium concentrations were significantly higher in adults (*n* = 6) than in juveniles (*n* = 4) in both liver (*U* = 0.0; *Z* = −2.452; *p* = 0.014) and kidney (*U* = 0.0; *Z* = −2.452; *p* = 0.014). In liver, Cu levels were significantly lower in adults than in juveniles (*U* = 0.0; *Z* = 2.452; *p* = 0.014), and in kidney, Cu levels were significantly higher in adults than in juveniles (*U* = 2.0; *Z* = −2.025; *p* = 0.043).

## Discussion

Our study revealed the presence of Cd, Pb, Cu, and Zn in liver and kidney of all sampled beavers. No relationship was observed between metal concentrations and the beavers’ sex. Although we acknowledge the limitations of the tests due to a reduced number of individuals in each group, these results were expected as the species present no sexual dimorphism and no partitioning in diet (Haarberg and Rosell 2006; Krojerová-Prokešová et al. 2010). Therefore, it was expected that both sexes be exposed similarly to environmental pollution.

Contrastingly, some metal concentrations were affected by the animals’ age. Higher liver and kidney Cd concentrations were found in adult beavers than in juveniles, indicating that this metal bioaccumulates over time (Parker and Hamr 2001; Bilandžić et al. 2010; Giżejewska et al. 2014). Cd levels in all six adult beavers exceed the maximum threshold of 1.0 μg/g in kidney recommended in farm animal edible tissues. In liver, however, only one adult female (F7) exceeded the recommended 0.5 μg/kg Cd limit (European Commission 2006). Such differences in Cd levels with age might be due to this metal accumulating over an animal’s entire life span, especially in kidney, as previously described in other species (e.g., red fox (*Vulpes vulpes*) and stone marten (*Martes foina*): Bilandžić et al. 2010; African grass owl (*Tyto capensis*): Ansara-Ross et al. 2013; penguins (*Pygoscelis* spp.): Jerez et al. 2013).

Average Cd content in kidney was similar to that observed in 2003 in beavers from Srokowo Forest Division, an uncontaminated area in northeastern Poland (Zalewski et al. 2012). However, our results were approximately four- to fivefold lower in liver and three- to fourfold lower in kidney than levels measured in earlier studies on beaver at unpolluted sites in Poland (Giżejewska et al. 2014), in Norway (Fimreite et al. 2001), and in a putatively contaminated former air base in Poland (Zalewski et al. 2012). Contrastingly, Cd concentrations in liver and kidney were higher than those reported in Canadian beavers (*Castor canadensis*) in habitats located 175 km from an ore smelter in Ontario, Canada (Wren 1984).

Cadmium concentration in wildlife tissue is attributed to its levels in the environment and additionally with animals’ age. Previous studies concerned with Cd content in unpolluted areas presented values that could be attributed to metal transport over long distances from the pollution source as well as increased mobility due to soil and water acidification (Wren 1984). Metal concentrations in animals’ tissues can be attributed to diet (Fimreite et al. 2001; Brekken and Steinnes 2004; Zalewski et al. 2012). Although metal concentrations in food sources typically consumed by beavers were not examined in the present study, it has been reported that beavers have a strong preference for aspen (*P. tremuloides*) and willow (*Salix* sp.), trees that accumulate high levels of Cd particularly on acidic soils that increase the mobility of heavy metals (Wren 1984). Cd is easily absorbed and accumulates even at low levels of chronic exposure (Nordberg et al. 2011b). Interspecific differences, intraspecific variations among populations, or physiological determinants of metal absorption and excretion can also contribute to the observed differences (Nordberg et al. 2011a).

All values of Pb found in beavers were below the 0.5 μg/g threshold recommended for both organs in farm animal edible tissues (European Commission 2006). Lead concentrations in kidney ranged from 0.052 μg/g (F9) to 0.142 μg/g (F10). Lead passes the placenta and young animals have a higher rate of absorption from the intestinal tract (Nordberg et al. 2011a). As this metal is not metabolically regulated in organisms, Pb concentrations in animal tissues are related to its presence in the ecosystem (Jerez et al. 2013). Soil intentionally or accidentally consumed by beavers could constitute an additional source of contamination as Pb can be bound in soil and bottom sediments and secondarily released (Rajaganapathy et al. 2011). Lead concentrations in our study were low compared to previous research (Table 3). This could be attributed to the absence of major industrial sites close to the sample site, seasonality, or individual variability in diet (Brekken and Steinnes 2004). Regardless, Pb threat to beavers appears low in the region.

We found significantly higher levels of Cu and Zn in liver than in kidney. Copper absorption is regulated by homeostatic mechanisms in the liver and is mainly excreted through the bile. Absorbed Zn is bound to plasma albumin and macroglobulins and distributed to the liver where it rapidly accumulates (Parker and Hamr 2001). Although Cu has higher affinity than Zn to metallothionein (low molecular weight metal-binding protein) which binds Cu and Zn at low levels of Cd (Hillis and Parker 1993; Nordberg et al. 2011a), hepatic metallothionein seems to have an important role in accumulation and storage of both Cu and Zn in the liver (Parker and Hamr 2001). Copper concentrations were higher in juvenile than in adult beavers in liver, indicating age-specific metabolism capabilities of this microelement (Nordberg et al. 2011a). Higher Cu levels in juveniles’ liver can be attributed to increased requirements for this element in growing organisms (Parker and Hamr 2001; Jerez et al. 2013). Our Cu values in both liver and kidney were higher than those in previous studies (Table 3). Copper as an essential microelement has effective regulation mechanisms of uptake and excretion, and beaver diet contains high amounts of Cu and Zn (Brekken and Steinnes 2004). Therefore, we assume that our values were at normal physiological levels (Nordberg et al. 2011a).

Zinc concentrations did not differ between organs and sex or age classes in our study. However, excluding beavers collected in the vicinity of the ore smelter in Ontario, Canada, where Zn concentrations reached 80.8 μg/g in the liver (Hillis and Parker 1993), we found Zn levels in liver higher than in previous studies (Table 3). In kidney, Zn levels were highly correlated to Cd levels regardless of the beaver age or sex. Zinc and cadmium have similar chemical properties, and they are usually found as a mineral combination in the environment (Nordberg et al. 2011b). Metallothionein can bind Cd, Cu, and Zn, and this protein will bind most of the Cd and store it in kidney. Exposure to low Cd levels may cause a redistribution of Zn in the organism, increasing Zn concentrations in kidney (Nordberg et al. 2011a). Higher kidney Zn concentrations were reported in beavers from Ontario (Hillis and Parker 1993). The highest Zn concentrations (liver 40.6 μg/g; kidney 27.9 μg/g) we found in adult female F7 could also possibly be attributed to a diet rich in this microelement (Brekken and Steinnes 2004).

## Conclusion and outlook

General metal concentrations in beaver tissues from northeastern Poland were similar to or lower than those found in other countries. This was expected as our study area was away from major industrial centers. However, the fact that high Cd concentrations and Pb were present in a putatively unpolluted area calls for a regular monitoring of environmental pollutants in agricultural and natural areas. Future investigation of trace elements in beaver tissues sampled from industrial regions is needed for comparison and to draw further conclusion about levels of contamination. Significant expansion of beaver populations in Poland contributes to growing human-beaver conflict, increasing the amount of compensation paid to farmers and landowners. Although beaver is currently not considered as a consumptive species in Poland, beaver is classified as a game species in six European countries (Belarus, Estonia, Lithuania, Latvia, Norway, and Sweden) as well as in Canada and Russia (Jankowska et al. 2005). Hunting beavers for consumption has been suggested as a tool for population management, and beaver consumption could occur in the near future in Poland. Research to ascertain tissues are safe from a toxicological point of view is therefore timely. In this context, the fact that Cd concentrations in adult beavers exceeded limits recommended in farm animal tissues warrants caution. Also, it would be necessary to investigate levels of persistent organic pollutants (POPs) in beaver tissues. POPs are hydrophobic and lipophilic persistent compounds that may accumulate along the food chain and can cause severe metabolic disturbance, such as endocrinal disturbance (Jones and de Voogt 1999). The presence of POPs in remote areas such as the Arctic is a major environmental issue (Brault et al. 2013). Consequently, we recommend future research and regular monitoring to identify the source of contaminants in the ecosystem and possible mitigation measures, and to ensure that contamination levels are within a safe range.
